# Estimating Benefits of Past, Current, and Future Reductions in Smoking Rates Using a Comprehensive Model With Competing Causes of Death

**DOI:** 10.5888/pcd9.110295

**Published:** 2012-07-05

**Authors:** Jeroen van Meijgaard, Jonathan E. Fielding

**Affiliations:** Author Affiliation: Jonathan E. Fielding, UCLA and Los Angeles County Department of Health, Los Angeles, California.

## Abstract

**Introduction:**

Despite years of declining smoking prevalence, tobacco use is still the leading preventable contributor to illness and death in the United States, and the effect of past tobacco-use control efforts has not fully translated into improvements in health outcomes. The objective of this study was to use a life course model with multiple competing causes of death to elucidate the ongoing benefits of tobacco-use control efforts on US death rates.

**Methods:**

We used a continuous-time life course simulation model for the US population. We modeled smoking initiation and cessation and 20 leading causes of death as competing risks over the life span, with the risk of death for each cause dependent on past and current smoking status. Risk parameters were estimated using data from the National Health Interview Survey that were linked to follow-up mortality data.

**Results:**

Up to 14% (9% for men, 14% for women) of the total gain in life expectancy since 1960 was due to tobacco-use control efforts. Past efforts are expected to further increase life expectancy by 0.9 years for women and 1.3 years for men. Additional reduction in smoking prevalence may eventually yield an average 3.4-year increase in life expectancy in the United States. Coronary heart disease is expected to increase as a share of total deaths.

**Conclusions:**

A dynamic individual-level model with multiple causes of death supports assessment of the delayed benefits of improved tobacco-use control efforts. We show that past smoking reduction efforts will translate into further increases in life expectancy in the coming years. Smoking will remain a major contributor to preventable illness and death, worthy of continued interventions.

## Introduction

Despite significant reductions in smoking prevalence nationally and changes in social norms surrounding tobacco use, tobacco use persists as the leading cause of preventable illness and death in the United States (1,2). From 2000 through 2004, one-fifth (45 million) of US adults smoked, resulting in an estimated 443,000 premature deaths and $193 billion in direct health care expenditures and productivity losses each year (1). Cigarette smoking is associated with or causally linked to myriad health conditions, including cardiovascular diseases; cancers of the lung, oral, and nasal cavities and of the esophagus, larynx, pancreas, kidney, and bladder; chronic obstructive pulmonary disease (COPD); and infertility, preterm birth, and low birth weight (3-6). In the United States, smoking annually causes more than 30% of all cancer deaths and more than 80% of lung cancer deaths (1,7).

Tobacco use control and prevention strategies (ie, education; comprehensive smoke-free policies; taxation of tobacco products; evidence-based, culturally targeted cessation approaches; and regulations on advertising, targeting, and promotion by tobacco companies) have successfully reduced the age-adjusted smoking prevalence rate among adults aged 18 or older by more than half, from 42.4% in 1965 to 19.3% in 2010 (8,9). Although the reductions in smoking prevalence that occurred over the last several decades have led to a substantial reduction in deaths from coronary heart disease attributed to smoking (10), lung cancer deaths have declined more slowly (7,11).

Health forecasting models have become more sophisticated with advances in computer technology, the increased availability of survey data, an improved understanding of the long-term consequences of lifestyle behaviors, and more complex concepts that are translated into models, reflecting a better understanding of interactions and disease processes (12). Smoking lends itself well to dynamic modeling because of the long delay between smoking and the manifestation of disease (eg, lung cancer), consistent data collected over many decades, and the unambiguous effect of smoking on multiple health problems.

Smoking-related health forecasts have been used to inform tobacco-use control strategies for different target populations (13) by enhancing understanding of the potential effect of specific policies and interventions on smoking rates (14). These models can predict short- and long-term changes in illness, death, life expectancy, quality-adjusted life years, female fertility, and health-care expenditures among smokers and the population overall (13,15-17). Full effects of smoking cessation can require up to 50 years to measure in individuals. Because cessation efforts translate slowly into declining smoking prevalence, it may take up to 100 years to see the full population effect of cessation efforts (14). This lag or delayed timing of benefits is rarely considered in models that estimate the magnitude of effect of smoking on outcomes.

Because morbidity and cause-specific mortality associated with smoking are affected by competing causes of death, a clearer picture of the effect of smoking on longevity would capture competing disease and injury causes of death and changes in competing risk factors for smoking-related diseases. Recent work has demonstrated that competing risks can be modeled to estimate the joint effect of smoking and obesity, the leading preventable causes of illness and death, on life expectancy and quality of life over a 15-year span (18). Although some models have examined the effect of smoking on cause-specific mortality (15), to our knowledge, no model has accounted for competing causes of death.

We addressed this gap by using the University of California, Los Angeles (UCLA) Health Forecasting Tool (www.health-forecasting.org) to estimate the effect of smoking on cause-specific mortality in the United States while accounting for competing causes of mortality. We estimated the life expectancy gains in the United States under various smoking scenarios. Life expectancy was used to standardize and interpret the magnitude of interventions on health outcomes (19,20).

The objective of this study was to use the UCLA Health Forecasting Tool to analyze the effect on US death rates of antismoking efforts and predict the nature and magnitude of future benefits.

## Methods

### Modeling framework

The simulation model is based on a dynamic and continuous-time framework previously developed for the UCLA Health Forecasting Model (12,21,22). Continuous-time modeling reduces the complexity of simulating multiple processes with many events that otherwise would explode the number of possible state transitions in a discrete-time model. The simulation framework provides an algorithm to generate individual lifetime histories starting at birth and using probabilities to determine which events happen during the life course. Smoking behavior is simulated by using initiation and cessation rates conditional on smoking status and age. Time since cessation is implicitly updated as the lifetime history is simulated. Mortality hazards are updated when age and smoking status change, including changes in the time since cessation.

### Smoking rates

We estimated smoking initiation and cessation rates using sequential cohorts from the National Health Interview Survey (NHIS). Initiation is modeled through young adulthood with a constant initiation rate through age 24, after which initiation is considered negligible (23). We estimated cumulative initiation through age 24 using the “Have you ever smoked” response on the NHIS survey and cessation using the change in prevalence of current smokers over a 5-year period to obtain the cessation rate of successful quitters. We estimated cessation rates for different age groups; the age cutoffs were selected after visual inspection of the smoothed cessation rates over the life span. We assumed negligible relapse after 5 years of smoking abstention. We calibrated initiation and cessation rates by using the simulation model to account for the decline in smoking prevalence from excess mortality among smokers. This approach yielded cumulative rates of initiation of 35% among women and 39% among men for the 1980 birth cohort (24-year-olds in 2004), with annual cessation rates of 4.2%, 3.1%, 2.5%, and 4.5% for women aged 15 to 27, 28 to 32, 33 to 47, and 48 or older, respectively, and 4.0%, 2.8%, 2.1%, and 6.0% for men aged 15 to 27, 28 to 32, 33 to 47, and 48 or older, respectively. These rates are consistent with observed rates reported elsewhere (24-27). The increase in cessation rates as age increases may be driven by health events, such as the onset of heart disease, of the individual or friends and relatives later in life (24,28,29). The smoking prevalence and time since last smoked, as generated by the model, were subsequently validated against the observed rates using NHIS data.

### Population and causes of death

We chose to simulate a representation of the 2004 US population, which gave us access to a robust data set that allowed estimating excess mortality related to tobacco use linked to follow-up data on cause-specific mortality. We created a synthetic population based on 2004 population and mortality data from the National Center for Health Statistics (NCHS) (30) and obtained cause-specific mortality rates for 2004 from the National Vital Statistics System. NCHS provides recodes for 39, 113, 130, and 358 selected causes of death, with varying degrees of specificity (31). We used the 39-cause list to identify the top 20 causes of death after excluding 4 nonspecific causes: “Other malignant neoplasms,” “Symptoms, signs, and abnormal clinical and laboratory findings, not elsewhere classified,” “All other diseases (Residual),” and “All other external causes.”

We estimated the parameters of our analysis by pooling health behavior data from NHIS for 1997 through 2004 and linked these with follow-up data on cause-specific mortality through the end of 2006 (32). Data for relative risks of smoking on cause-specific mortality are available for select causes and populations (33) but not for each of the 20 leading causes of death separately for men and women. Therefore, we estimated a Cox proportional hazards model for each cause of death to obtain relative risk parameters of smoking (never, current, former [0–4 y, 5–9 y, 10–19 y, or ≥20 y since quit]) on cause-specific mortality. The estimates were stratified by sex, and the baseline hazards were stratified by age (5-year age categories). Relative risk estimates were adjusted for race/ethnicity (6 categories), education (less than high school, high school diploma, more than high school), income (<100%, 100%–400%, >400% of the federal poverty level), body mass index (BMI, continuous), physical activity (metabolic equivalent time, continuous), and alcohol consumption (no alcohol, 0–2 drinks/d, >2 drinks/d).

### Simulation scenarios

We simulated 2 sets of scenarios. The first set of scenarios estimated smoking attributable deaths for the 2004 population and validated the relative risk of smoking on all-cause mortality by comparing our estimates with other studies. To estimate smoking-attributable deaths, we ran the simulation model for a 2004 reference scenario, applying 2004 smoking rates to 40 million simulated individuals reflecting the 2004 population. Next, we ran the counterfactual scenario with all relative risks of smoking on mortality set to 1, assuming that smoking has no effect on mortality. We compared our estimates of smoking-attributable deaths with estimates from the Centers for Disease Control and Prevention (CDC) (1) to validate our model.

The second set of scenarios estimated the effect of past, current, and future changes in initiation and cessation rates using a cohort analysis. We used 2004 mortality rates throughout the life course, similar to life table calculations, and compared mortality and life expectancy in a birth cohort followed from birth to death. We held initiation and cessation rates fixed at levels specified in each scenario. We simulated cohorts of 4 million individuals in each scenario, by using different assumptions about smoking initiation and cessation rates to estimate the timing of changes in smoking initiation and cessation on mortality (Box). Comparing scenarios 3 and 6, for example, yields the difference in mortality, life expectancy, and distribution of causes of death between never smokers and always smokers (if viewed from the individual perspective, the probabilistic outcome of death with continuous lifelong smoking vs never smoking). We compared age-adjusted mortality rates and life expectancy with the reference scenario to estimate past and potential future gains from tobacco-use control efforts.

**Box. Smoking Initiation and Cessation Scenarios**

**Table:** 

**Scenario**	**Rationale**
**2004 Analysis**	
1. Reference calibrated to 2004	Used as a reference to compare alternative scenarios
2. Counterfactual where smoking has no effect on mortality	Used to calculate smoking attributable deaths by comparing to the reference scenario
**Cohort Analysis**	
3. 100% initiation and 0% cessation for all adult men and women	Used to generate distribution of causes of death for “always smokers”
4. 55% initiation for women/80% initiation for men and cessation half of 2004 rates (described in text)	Used to generate distribution of causes of death for cohort assuming smoking rates observed in the 1940–1950s before public health action to reduce smoking prevalence (to estimate the effect of antismoking public health programs)
5. 2004 initiation and cessation rates (described in text)	Used to generate distribution of causes of death for cohort assuming 2004 initiation and quit rates
6. 0% initiation	Used to generate distribution of causes of death for “never smokers”

### Timing of benefits

Smoking-related deaths occur among people of all ages. Gains in life expectancy occur across a significant portion of the life span and not just later in life. We estimated expected gains at the individual level for men and women by repeated simulation of individuals quitting smoking at various ages and comparing the total remaining life years to those of lifetime smokers. These individual-level gains were aggregated over a simulated cohort for each scenario, yielding gains in life years across the lifespan for the entire cohort. We calculated gains in life years relative to scenario 4, which used initiation and cessation rates from the 1950s, to calculate past and future gains from reductions in smoking. To calculate gains in life years for the 2004 population, we applied age-specific 2004 mortality rates to a standard cohort, similar to life expectancy calculations.

## Results

### Model validation

We estimated that 18.9% of adult women and 23.1% of adult men were current smokers in 2004, as compared with observed rates of 18.5% and 23.4%, respectively (34). We estimated smoking-attributable deaths at 420,000 in 2004 (Table 1), which is comparable to CDC estimates of 443,000 deaths annually from 2000 through 2004 (1). We found that 15% of all deaths among women and 20% of all deaths among men were attributable to smoking. Most of the deaths avoided derive from a reduction in cancer of the trachea, bronchus, or lung (27%) and chronic lower respiratory diseases (21%). The data presented in Table 2, simulated smoking rates, life expectancy, and mortality by cause of death for 2004, are a reference to compare the 4 scenarios. In 2004, nearly half of adult men and approximately 37% of adult women had smoked at some point in their adult life; life expectancy was 75.1 years for men and 80.2 years for women.

**Table 1:** Estimated Deaths Avoided in the Absence of Smoking, Causes of Death, Simulation Model, 2004 US Population

Cause of Death	Deaths Avoided (in Thousands), n (% Reduction)		Age-Adjusted Mortality (per 100,000), n (% Total Mortality)	

	Female	Male	Female	Male
Ischemic heart diseases	25 (15)	41 (17)	103 (18)	172 (21)
Cancer of trachea, bronchus, and lung	49 (70)	66 (71)	12 (2)	22 (3)
Chronic lower respiratory diseases	47 (72)	40 (66)	10 (2)	18 (2)
All other causes	60 (7)	92 (11)	277 (48)	354 (44)
Total	181 (15)	239 (20)	579 (100)	808 (100)

**Table 2:** Life Expectancy and Mortality, by Cause of Death, United States, 2004^a^

Life Expectancy	Female		Male	
Current smokers (adults ≥18), %	18.9		23.1	
Former smokers (adults ≥18), %	18.2		25.7	
Life expectancy, y	80.2		75.1	

**Cause of Death**	**Total Deaths (in Thousands)**	**Age-Adjusted (Per 100,000 Population)**	**Total Deaths (in Thousands)**	**Age-Adjusted (Per 100,000 Population)**

All	1,222	688	1,193	1,002
Ischemic heart diseases	219	118	236	205
Other heart diseases (no CHD)	94	51	75	67
Cancer of trachea, bronchus, and lung	70	42	92	73
Cerebrovascular diseases	92	50	59	53
Chronic lower respiratory diseases	65	37	61	53
Diabetes mellitus	38	22	36	29
Unspecified accidents and adverse effects	28	17	42	32
Alzheimer’s disease	47	24	20	20
Influenza and pneumonia	33	18	27	25
Cancer of colon, rectum, and anus	27	16	26	22
Motor vehicle accidents	15	10	31	22
Nephritis, nephrotic syndrome, and nephrosis	22	12	21	19
Cancer of breast	41	25	0	0
Intentional self-harm	7	5	26	18
Hypertensive heart disease	17	9	15	12
Cancer of pancreas	16	9	16	12
Cancer of prostate	NA	NA	29	27
Chronic liver disease and cirrhosis	9	6	18	13
Cancer of urinary tract	9	5	17	14
Cancer of cervix uteri, corpus uteri, and ovary	25	15	NA	NA
All other causes	348	198	348	286

Abbreviation: NA, not applicable.

^a^ Numbers in the table are from model output for 2004 after calibration with vital statistics data from the National Center for Health Statistics.

### Life expectancy benefits

Table 3 summarizes estimates of the effect of smoking initiation and cessation on smoking prevalence and mortality outcomes for scenarios 3 through 6. Smoking prevalence is age-adjusted to the 2004 US population by sex to facilitate comparison of the cohort scenarios with the reference scenario (scenario 1). Initiation and cessation rates in the 1940s and 1950s, scenario 4, result in a lifetime smoking prevalence of 33% for women and 48% for men; life expectancy is reduced to 79.6 years for women and 73.9 years for men.

**Table 3:** Life Expectancy, Age-Adjusted Mortality, and Causes of Deaths in a Cohort Using Different Smoking Rates, United States^a^

Cause of Death/Life Expectancy/Age-Adjusted Mortality	Scenario 3: All Smoking		Scenario 4: 1940s-1950s Rates		Scenario 5: 2004 Rates		Scenario 6: No Smoking	

	Female	Male	Female	Male	Female	Male	Female	Male
Current adult smokers,^b^ %	100	100	32.9	47.9	14.4	16.4	0	0
Former adult smokers,^b^ %	0	0	20.2	30.3	19.2	21.2	0	0
Life expectancy, y	74.9	70.1	79.6	73.9	81.1	76.4	82.3	78.0
Age-adjusted mortality^c^	1,176	1,644	725	1,080	646	896	579	805
**Distribution of Deaths by Cause, %**								
Ischemic heart diseases	17.9	21.8	18.9	21.0	19.5	21.9	20.5	22.8
Other heart diseases (no CHD)	4.4	6.1	7.9	6.6	8.9	7.5	9.9	8.0
Cancer of trachea, bronchus, and lung	14.4	14.7	6.1	8.7	3.5	4.7	1.8	2.6
Cerebrovascular diseases	7.6	3.2	7.8	5.1	8.2	6.3	8.7	7.0
Chronic lower respiratory diseases	11.3	10.0	6.2	6.2	4.3	3.9	1.7	2.4
Diabetes mellitus	1.9	1.8	2.9	2.7	3.1	3.2	3.4	3.5
Unspecified accidents and adverse effects	3.7	2.9	2.1	2.9	1.9	3.0	1.8	3.0
Alzheimer’s disease	2.0	1.2	4.1	1.9	4.7	2.5	5.3	2.8
Influenza and pneumonia	2.3	2.8	2.9	2.7	3.1	2.9	3.4	3.0
Cancer of colon, rectum, and anus	1.8	2.1	2.1	2.2	2.1	2.3	2.1	2.5
Motor vehicle accidents	0.8	1.3	0.8	1.6	0.8	1.7	0.8	1.8
Nephritis, nephrotic syndrome, and nephrosis	1.1	1.1	1.8	1.8	2.0	2.2	2.1	2.4
Cancer of breast	1.4	0	2.8	0	3.0	0	3.1	0
Intentional self-harm	0.8	1.8	0.4	1.7	0.3	1.3	0.2	1.1
Hypertensive heart disease	0.8	0.7	1.4	1.1	1.6	1.3	1.8	1.4
Cancer of pancreas	1.4	0.8	1.3	1.2	1.2	1.4	1.2	1.5
Cancer of prostate	NA	1.6	NA	2.6	NA	3.3	NA	3.7
Chronic liver disease and cirrhosis	0.9	1.3	0.6	1.2	0.6	1.0	0.6	1.0
Cancer of urinary tract	0.4	1.5	0.7	1.5	0.7	1.2	0.8	0.9
Cancer of cervix uteri, corpus uteri, and ovary	1.3	NA	1.8	NA	1.9	NA	2.0	NA
All other causes	23.0	23.3	27.5	27.4	28.5	28.4	28.9	28.6

Abbreviation: CHD, coronary heart disease; NA, not applicable.

^a^ Cohorts simulated from birth to death to calculate eventual cause of death for alternative scenarios.

^b^ Aged ≥18 y, age-adjusted.

^c^ Per 100,000 2004 population.

Comparing scenario 4 with the reference scenario shows that changes in initiation and cessation rates since 1960 have resulted in a life expectancy increase of 0.6 years for women and 1.2 years for men. Moreover, at 2004 initiation and cessation rates (scenario 5), an additional 0.9 years for women (life expectancy, 81.1 y) and 1.3 years for men (life expectancy, 76.4 y) may be realized in the future, because 2004 initiation and cessation rates will continue to yield reductions in current smokers and ever smokers and their related mortality (scenario 5 vs reference). Fully eliminating tobacco use in the population (scenario 6) would yield an increase in life expectancy of 1.2 years for women and 1.6 years for men, or 82.3 and 78.0 years, respectively.

### Causes of mortality

Although overall age-adjusted mortality declines with reductions in smoking prevalence, trends vary by disease. Reductions in smoking prevalence lead to a substantial reduction in deaths from lung cancer and COPD. However, although smoking increases the risk of coronary heart disease, heart disease as a percentage of total deaths is higher for never smokers than for always smokers (scenario 3 compared with scenario 6). In fact, the share of total deaths held by ischemic heart disease (IHD) has risen and is expected to continue to increase if smoking prevalence declines further (Table 3).

### Timing of benefits

**Figure 1:**
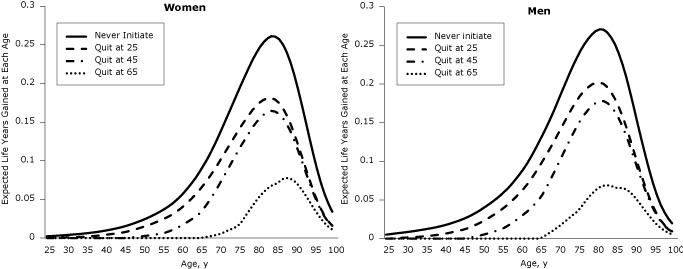
Expected gain in years of life across the lifespan by age of quitting, relative to a lifetime smoker, by sex. After quitting smoking, individuals are more likely to be alive at every age after the quit age. The largest gain is around age 80, but gains are smaller for those who quit later in life.

**Figure 2:**
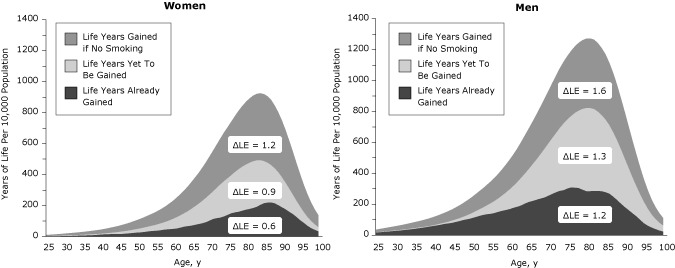
Gains in years of life relative to mid-1900s initiation and cessation rates, by age and sex. Lower initiation and cessation rates have yielded additional life years in the population at all ages (area under the curve is the gain in life expectancy (ΔLE), and additional gains are expected if initiation and cessation rates stay at 2004 levels.

Longevity gains of quitters relative to lifetime smokers were recorded across the lifespan (Figure 1). Using a cohort of 10,000 people at birth, we plotted years of life gained across the life span (Figure 2). The life years already gained is the difference between the years of life for the 2004 population (reference scenario) and scenario 4. The life years yet to be gained if 2004 initiation and cessation rates persist are the difference between scenario 5 and the reference scenario, and the life years that may be gained if no one ever smoked is the difference between scenario 6 and scenario 5. The area under the curve is equal to the gain in life expectancy (ΔLE). Substantial gains have already been realized during the life course (life expectancy gain of 0.6 y for women and 1.2 y for men), and additional gains will occur mostly at older ages if initiation and cessation rates stay at 2004 levels (additional life expectancy gain of 0.9 y for women and 1.3 y for men) (Figure 2). The largest gains in years of life across the lifespan may be realized if all individuals in the cohort remain never smokers, providing an additional gain in life expectancy of 1.2 years for women and 1.6 years for men (scenario 6).

**Table:** 

Age, y	Expected Life Years Gained at Each Age							

	Women				Men			

	Never Initiate	Quit at 25	Quit at 45	Quit at 65	Never Initiate	Quit at 25	Quit at 45	Quit at 65
**25**	0.002	0	0	0	0.005	0	0	0
**30**	0.004	0.001	0	0	0.008	0.001	0	0
**35**	0.006	0.002	0	0	0.012	0.003	0	0
**40**	0.009	0.004	0	0	0.017	0.006	0	0
**45**	0.015	0.007	0	0	0.025	0.011	0	0
**50**	0.023	0.014	0.002	0	0.038	0.021	0.004	0
**55**	0.035	0.023	0.006	0	0.056	0.037	0.012	0
**60**	0.053	0.038	0.015	0	0.083	0.059	0.027	0
**65**	0.085	0.062	0.031	0	0.124	0.092	0.050	0
**70**	0.131	0.096	0.067	0.004	0.177	0.135	0.095	0.015
**75**	0.187	0.137	0.111	0.015	0.234	0.179	0.145	0.034
**80**	0.239	0.171	0.149	0.048	0.269	0.202	0.176	0.063
**85**	0.261	0.179	0.164	0.070	0.252	0.181	0.164	0.066
**90**	0.211	0.135	0.126	0.074	0.169	0.113	0.104	0.055
**95**	0.108	0.061	0.058	0.039	0.072	0.042	0.039	0.024
**100**	0.034	0.016	0.015	0.011	0.020	0.009	0.009	0.006

**Table:** 

Age, y	Years of Life Gained Per 10,000 at Each Age					

	Women			Men		

	Years of Life Already Gained	Years of Life Yet to be Gained	Years of Life Gained if No Smoking	Years of Life Already Gained	Years of Life Yet to be Gained	Years of Life Gained if No Smoking
**25**	0	4.8	2.6	17.4	1.6	15.7
**30**	0.9	3.9	6.7	28.0	3.5	25.4
**35**	4.9	3.7	12.2	41.2	4.9	36.7
**40**	8.4	5.5	18.1	60.2	4.9	51.1
**45**	15.3	6.1	27.1	81.0	15.0	70.9
**50**	18.2	16.0	40.8	110.3	32.4	100.9
**55**	28.0	26.0	60.3	138.7	68.8	138.9
**60**	39.8	42.6	85.7	169.7	129.3	190.3
**65**	53.3	73.5	124.4	216.2	215.3	260.5
**70**	76.8	119.8	182.8	255.5	338.3	335.6
**75**	114.7	179.2	260.4	302.6	447.4	408.2
**80**	151.7	246.3	343.1	285.2	538.5	447.9
**85**	179.2	298.9	412.6	278.6	469.8	418.8
**90**	220.3	257.8	427.0	183.2	314.4	297.4
**95**	177.1	171.1	335.5	78.0	139.2	142.3
**100**	89.8	69.5	172.4	24.2	39.4	46.9
**Change in Life Expectancy**	0.6	0.9	1.2	1.2	1.3	1.6

## Discussion

Our simulation model is a dynamic tool to estimate health effects of various scenarios, taking into account the timing of smoking initiation, cessation, and the effect on health outcomes. We can evaluate what may have happened if smoking behavior had not changed and estimate what could be attained with further tobacco-use control efforts. We found that, as of 2004, reductions in smoking prevalence resulted in life expectancy gains equal to nearly 9% of the total gain in female life expectancy and 14% of the total gain in male life expectancy from 1960 to 2004. However, at current initiation and cessation rates, additional life expectancy gains approximately equal to the total observed gains from 1995 through 2004 are expected (35). The magnitude of these gains and the potential for additional gains if smoking rates can be reduced further underscore the importance of continuing tobacco-use control efforts.

The simulation also helps assess the distribution of gains across the lifespan and how the fractions of mortality attributable to various diseases may change as smoking prevalence is reduced. For example, as a share of total deaths, IHD is expected to increase, despite a decline in smoking rates and a decline in age-adjusted IHD mortality, reflecting a shift in mortality away from lung cancer and COPD to IHD and other causes of death. Causes of death minimally affected by smoking, such as injuries, or occurring primarily at older ages, such as Alzheimer’s disease, will also increase their share of total deaths.

We have inherent limitations in our modeling approach. First, our model treats people separately from their environment. Therefore, passive smoking and effects of air pollution are ignored. Similarly ignored are intergenerational effects, including how smoking by pregnant mothers affects the health and mortality risk of infants and how parents who smoke are more likely to have children who eventually initiate smoking. Furthermore, smoking intensity was not modeled in the simulation. This exclusion may bias effect estimates if ongoing cessation efforts have also changed smoking intensity. However, our estimates of the benefits of reduced smoking initiation and increased cessation are likely conservative with the exclusion of passive smoking, intergenerational effects, and decline in smoking intensity (8). Moreover, by focusing on smoking-attributable mortality, we omit the quality-of-life benefits from reductions in smoking-related morbidity.

Although this study incorporates competing causes of death, it did not include comorbidities and competing behavioral risks for illness and death, despite their potential relevance. For example, mental illness is associated with higher smoking prevalence and other unhealthy behaviors as well as increased mortality (36,37). Also, obesity differentially affects smoking-related diseases (18), and current increases in obesity prevalence are likely to further increase cardiovascular mortality relative to lung cancer and COPD mortality.

Our model can help inform future public health campaigns and assist in prioritizing scarce resources. Future work should focus on adding additional health risk factors, such as obesity or other morbidities, to better understand how reductions in smoking prevalence will reduce and shift the burden of disease. Moreover, expanding the framework to include passive smoking and intergenerational effects would better capture the full benefits of reductions in smoking prevalence, and stratification by race/ethnicity would provide insight into causes of health disparities.

## References

[1] Centers for Disease Control and Prevention. Smoking-attributable mortality, years of potential life lost, and productivity losses — United States, 2000-2004. MMWR Morb Mortal Wkly Rep 2008;57(45):1226-8. 19008791

[2] Mokdad AH , Marks JS , Stroup DF , Gerberding JL . Actual causes of death in the United States, 2000. JAMA 2004;291(10):1238-45. 10.1001/jama.291.10.1238 15010446

[3] Agrawal A , Scherrer JF , Grant JD , Sartor CE , Pergadia ML , Duncan AE , The effects of maternal smoking during pregnancy on offspring outcomes. Prev Med 2010;50(1-2):13-8. 10.1016/j.ypmed.2009.12.009 20026103 PMC2813884

[4] Cornfield J , Haenszel W , Hammond EC , Lilienfeld AM , Shimkin MB , Wynder EL . Smoking and lung cancer: recent evidence and a discussion of some questions. Int J Epidemiol 2009;38(5):1175-91. 10.1093/ije/dyp289 19773415

[5] The health consequences of smoking: a report of the Surgeon General. Atlanta (GA): US Department of Health and Human Services; 2004.

[6] White WB . Smoking-related morbidity and mortality in the cardiovascular setting. Prev Cardiol 2007;10(2 Suppl 1)1-4. 10.1111/j.1520-037X.2007.06050.x 17396061

[7] Shopland DR , Eyre HJ , Pechacek TF . Smoking-attributable cancer mortality in 1991: is lung cancer now the leading cause of death among smokers in the United States? J Natl Cancer Inst 1991;83(16):1142-8. 10.1093/jnci/83.16.1142 1886147

[8] Centers for Disease Control and Prevention. Vital signs: current cigarette smoking among adults aged ≥18 years — United States, 2005–2010. MMWR Morb Mortal Wkly Rep 2011;60(35):1207-12. 21900875

[9] Giovino GA , Schooley MW , Zhu BP , Chrismon JH , Tomar SL , Peddicord JP , Surveillance for selected tobacco-use behaviors — United States, 1900-1994. MMWR CDC Surveill Summ 1994;43(3):1-43. 7969014

[10] Ford ES , Ajani UA , Croft JB , Critchley JA , Labarthe DR , Kottke TE , Explaining the decrease in US deaths from coronary disease, 1980-2000. N Engl J Med 2007;356(23):2388-98. 10.1056/NEJMsa053935 17554120

[11] Thun MJ , Jemal A . How much of the decrease in cancer death rates in the United States is attributable to reductions in tobacco smoking? Tob Control 2006;15(5):345-7. 10.1136/tc.2006.017749 16998161 PMC2563648

[12] van Meijgaard J , Fielding JE , Kominski GF . Assessing and forecasting population health: integrating knowledge and beliefs in a comprehensive framework. Public Health Rep 2009;124(6):778-89. 19894419 10.1177/003335490912400604 PMC2773940

[13] Tengs TO , Osgood ND , Lin TH . Public health impact of changes in smoking behavior: results from the Tobacco Policy Model. Med Care 2001;39(10):1131-41. 10.1097/00005650-200110000-00010 11567175

[14] Jha P . Avoidable global cancer deaths and total deaths from smoking. Nat Rev Cancer 2009;9(9):655-64. 10.1038/nrc2703 19693096

[15] Akushevich I , Kravchenko JS , Manton KG . Health-based population forecasting: effects of smoking on mortality and fertility. Risk Anal 2007;27(2):467-82. 10.1111/j.1539-6924.2007.00898.x 17511712

[16] Hurley SF , Matthews JP . The Quit Benefits Model: a Markov model for assessing the health benefits and health care cost savings of quitting smoking. Cost Eff Resour Alloc 2007;5:2. 10.1186/1478-7547-5-2 17241477 PMC1796848

[17] Wang H , Preston SH . Forecasting United States mortality using cohort smoking histories. Proc Natl Acad Sci U S A 2009;106(2):393-8. 10.1073/pnas.0811809106 19124775 PMC2613940

[18] Stewart ST , Cutler DM , Rosen AB . Forecasting the effects of obesity and smoking on US life expectancy. N Engl J Med 2009;361(23):2252-60. 10.1056/NEJMsa0900459 19955525 PMC4394736

[19] Wright JC , Weinstein MC . Gains in life expectancy from medical interventions — standardizing data on outcomes. N Engl J Med 1998;339(6):380-6. 10.1056/NEJM199808063390606 9691106

[20] Parrish RG . Measuring population health outcomes. Prev Chronic Dis 2010;7(4):A71. 20550829 PMC2901569

[21] Will BP , Berthelot JM , Nobrega KM , Flanagan W , Evans WK . Canada’s Population Health Model (POHEM): a tool for performing economic evaluations of cancer control interventions. Eur J Cancer 2001;37(14):1797-804. 10.1016/S0959-8049(01)00204-0 11549434

[22] Shi L , van Meijgaard J , Fielding J . Forecasting diabetes prevalence in California: a microsimulation. Prev Chronic Dis 2011;8(4):A80.http://www.cdc.gov/pcd/issues/2011/jul/10_0177.htm. 21672404 PMC3136987

[23] National household survey of drug abuse: advance report no. 18. Washington (DC): US Department of Health and Human Services, SAMHSA, Office of Applied Studies; 1991.

[24] Hyland A , Li Q , Bauer JE , Giovino GA , Steger C , Cummings KM . Predictors of cessation in a cohort of current and former smokers followed over 13 years. Nicotine Tob Res 2004;6(Suppl 3)S363-9. 10.1080/14622200412331320761 15799599

[25] Centers for Disease Control and Prevention. Smoking cessation during previous year among adults — United States, 1990 and 1991. MMWR Morb Mortal Wkly Rep 1993;42(26):504-7. 8515740

[26] DeCicca P , Kenkel DS , Mathios AD ; National Bureau of Economic Research. Cigarette taxes and the transition from youth to adult smoking smoking initiation, cessation, and participation. In: NBER working paper series no. 14042. Cambridge (MA): National Bureau of Economic Research; 2008. 10.1016/j.jhealeco.2008.02.008 18513811

[27] Hatziandreu EJ , Pierce JP , Lefkopoulou M , Fiore MC , Mills SL , Novotny TE , Quitting smoking in the United States in 1986. J Natl Cancer Inst 1990;82(17):1402-6. 10.1093/jnci/82.17.1402 2388290

[28] Reichert VC , Folan P , Bartscherer D , Jacobsen D , Fardellone C , Metz C , A comparison study of older smokers vs younger smokers being treated for tobacco dependence. Chest 2007;132(4):489s-489s.

[29] Sachs-Ericsson N , Schmidt NB , Zvolensky MJ , Mitchell M , Collins N , Blazer DG . Smoking cessation behavior in older adults by race and gender: the role of health problems and psychological distress. Nicotine Tob Res 2009;11(4):433-43. 10.1093/ntr/ntp002 19299410 PMC2670367

[30] Estimates of the July 1, 2000-July 1, 2004, United States resident population from the Vintage 2004 postcensal series by year, county, age, sex, race, and Hispanic origin, prepared under a collaborative arrangement with the US Census Bureau. Hyattsville (MD): National Center for Health Statistics; 2005.

[31] Heron M . Deaths: leading causes for 2004. Natl Vital Stat Rep 2007;56(5):1-95. 18092547

[32] Data file documentation, National Health Interview Survey 1997-2004 and linked mortality files (machine readable data file and documentation). Hyattsville (MD): National Center for Health Statistics; 2010.

[33] Kenfield SA , Wei EK , Rosner BA , Glynn RJ , Stampfer MJ , Colditz GA . Burden of smoking on cause-specific mortality: application to the Nurses’ Health Study. Tob Control 2010;19(3):248-54. 10.1136/tc.2009.032839 20501499 PMC3050007

[34] Centers for Disease Control and Prevention. Cigarette smoking among adults — United States, 2004. MMWR Morb Mortal Wkly Rep 2005;54(44):1121-4. 16280969

[35] Health, United States, 2010: with special feature on death and dying. Hyattsville (MD): National Center for Health Statistics; 2011. 21634072

[36] Lasser K , Boyd JW , Woolhandler S , Himmelstein DU , McCormick D , Bor DH . Smoking and mental illness: a population-based prevalence study. JAMA 2000;284(20):2606-10. 10.1001/jama.284.20.2606 11086367

[37] Lawrence D , Coghlan R . Health inequalities and the health needs of people with mental illness. N S W Public Health Bull 2002;13(7):155-8. 10.1071/NB02063 12451410

